# Pan-CDK inhibition augments cisplatin lethality in nasopharyngeal carcinoma cell lines and xenograft models

**DOI:** 10.1038/s41392-018-0010-0

**Published:** 2018-04-13

**Authors:** Nicholas L. Syn, Pei Li Lim, Li Ren Kong, Lingzhi Wang, Andrea Li-Ann Wong, Chwee Ming Lim, Thomas Kwok Seng Loh, Gerhard Siemeister, Boon Cher Goh, Wen-Son Hsieh

**Affiliations:** 1 Cancer Science Institute of Singapore, National University of Singapore, Singapore, Singapore; 2 Department of Haematology-Oncology, National University Cancer Institute, Singapore, Singapore; 3 Department of Pharmacology, Yong Loo Lin School of Medicine, National University Health System, Singapore, Singapore; 4 Department of Otolaryngology-Head and Neck Surgery, National University Health System, Singapore, Singapore; 5 Drug Discovery, TRG Oncology, Bayer Pharma AG, Berlin, Germany

## Abstract

In addition to their canonical roles in regulating cell cycle transition and transcription, cyclin-dependent kinases (CDKs) have been shown to coordinate DNA damage response pathways, suggesting a rational pairing of CDK inhibitors with genotoxic chemotherapeutic agents in the treatment of human malignancies. Here, we report that roniciclib (BAY1000394), a potent pan-CDK inhibitor, displays promising anti-neoplastic activity as a single agent and potentiates cisplatin lethality in preclinical nasopharyngeal carcinoma (NPC) models. Proliferation of the NPC cell lines HONE-1, CNE-2, C666-1, and HK-1 was effectively curbed by roniciclib treatment, with IC_50_ values between 11 and 38 nmol/L. These anticancer effects were mediated by pleiotropic mechanisms consistent with successful blockade of cell cycle CDKs 1, 2, 3, and 4 and transcriptional CDKs 7 and 9, ultimately resulting in arrest at G1/S and G2/M, downregulation of the transcriptional apparatus, and repression of anti-apoptotic proteins. Considerably enhanced tumor cell apoptosis was achieved following combined treatment with 10 nmol/L roniciclib and 2.0 μmol/L cisplatin; this combination therapy achieved a response over 250% greater than either drug alone. Although roniciclib chemosensitized NPC cells to cisplatin, it did not sensitize untransformed (NP69) cells. The administration of 0.5 mg/kg roniciclib to BALB/c xenograft mice was well tolerated and effectively restrained tumor growth comparable to treatment with 6 mg/kg cisplatin, whereas combining these two agents produced far greater tumor suppression than either of the monotherapies. In summary, these data demonstrate that roniciclib has strong anti-NPC activity and synergizes with cisplatin chemotherapy at clinically relevant doses, thus justifying further evaluation of this combinatorial approach in clinical settings.

## Cancer: Cell cycle–blocking drug active against throat cancer

Nasopharyngeal carcinoma (NPC) is an uncommon malignancy arising from the nasopharynx epithelium, and is endemic to east and southeast parts of Asia where they account for 70% of worldwide incidence. Researchers from the Cancer Science Institute of Singapore examined the anti-tumor effects of roniciclib**—**a small-molecule drug that blocks a family of enzymes known as cyclin-dependent kinases (CDKs) which are classically involved in cell cycle progression and transcription—in cell lines and mouse models of nasopharyngeal carcinoma. Because CDK/cyclin complexes have a putative role in DNA repair, roniciclib was combined with cisplatin, a DNA-damaging agent which is currently used in chemotherapy of NPC. The authors found that roniciclib had potent anti-NPC effects when given alone, whereas the combination of roniciclib and cisplatin proved to be highly synergistic and restrained tumor growth to a greater extent than either drugs given alone. Interestingly, roniciclib appeared to selectively enhance the anti-cancer effects of cisplatin in cancerous cells while this “chemo-sensitizing” phenomenon was not seen in non-cancerous cells, suggesting that giving both drugs together could improve the effectiveness of standard chemotherapy without incurring additional toxicities. These findings suggest that roniciclib should be evaluated clinically in patients with NPC.

## Introduction

The cyclin-dependent kinases (CDKs) are classically a family of serine/threonine kinases that orchestrate the precise spatiotemporal control of a multitude of biological functions related to cell cycle progression.^1,2^ In addition, they have been demonstrated to coordinate DNA damage responses by recognizing aberrant DNA structures and activating checkpoint and repair mechanisms.^3,4^ These regulatory circuits are co-opted by virtually all malignancies to promote their own growth and survival, rendering CDKs compelling targets for pharmacological inhibition.^5^

Roniciclib (BAY1000394) is a novel pan-CDK inhibitor with potent antiproliferative activity at low nanomolar concentrations. Siemeister et al. previously demonstrated roniciclib-mediated inhibition of cell cycle CDKs 1, 2, 3, and 4 and transcriptional CDKs 7 and 9 with IC_50_ values between 5 and 25 nmol/L in a broad spectrum of cancer cell lines.^6^ Two parallel first-in-human phase I studies have also recently concluded and have reported acceptable tolerability profiles and encouraging efficacy signals in advanced malignancies including small-cell lung cancer (SCLC), non-small cell lung cancer (NSCLC), and ovarian cancer.^7–9^ The pharmacological basis for simultaneously targeting multiple rather than individual CDKs lies in their well-known ability to compensate for one another’s functions.^10^ For example, dual blockade of the DNA damage response CDKs 1 and 2, but not CDK1 alone, is required to undermine DNA end resection,^11^ while CDK4/6 may fully assume the role of phosphorylating retinoblastoma (Rb), thus releasing the transcription factor E2F in order to sustain cancer cell proliferation in the absence of CDK2.^12^

To date, no preclinical or clinical studies have examined the effects of roniciclib in nasopharyngeal carcinoma (NPC), a unique epithelial malignancy which has an extremely skewed ethnogeographical distribution and is endemic to parts of Asia and Africa.^13^ Our prior experience with seliciclib, a cell cycle modulator, yielded promising results; 7 of 14 patients with locally advanced disease showed tumor regression,^14^ implying that NPC is amenable to CDK inhibition. Although advances in radiotherapy technology and broader application of chemotherapy have led to declining mortality over the past decade, survival rates have plateaued, and newer therapeutic approaches are urgently needed. A rational combination strategy consisting of roniciclib plus standard genotoxic chemotherapeutic agents may help to fill this therapeutic void.

This study was conducted to appraise the antitumor activity of roniciclib in NPC cell lines and xenograft models both as a single therapy and in combination with cisplatin, a DNA-damaging chemotherapeutic agent often used concurrently with radiotherapy in the treatment of locoregionally advanced and metastatic NPC.^13^ Here, we report that roniciclib alone, administered at clinically achievable concentrations, displays marked anti-NPC activity and synergistically increases cisplatin lethality both in vitro and in vivo. Together, these experiments provide a rationale for further clinical studies of roniciclib in NPC.

## Materials and methods

### Cell culture and drugs

We cultured human nasopharyngeal carcinoma cell lines (HONE-1, CNE-2, C666-1, and HK-1) and human colorectal carcinoma cell lines (HCT-116-WT and HCT-116-p53^−/−^) in RPMI (Sigma-Aldrich, St Louis, MO, USA) + 10% fetal bovine serum at 37 °C in 5% CO_2_. All cell lines were authenticated in 2013 by short tandem repeat analysis using the GenePrint 10 System (Promega). The immortalized nasopharyngeal epithelial cell line NP69 was maintained in keratinocyte serum-free medium (Invitrogen) supplemented with 5% heat-inactivated fetal calf serum, 25 μg/mL bovine pituitary extract, and 0.2 ng/mL recombinant epidermal growth factor according to the manufacturer’s recommendations. Roniciclib and cisplatin were obtained from Bayer Pharma AG and Pfizer Inc., respectively. Cisplatin was used at 50 mg/mL in all experiments. For in vitro experiments, a stock roniciclib solution was prepared at 10 mmol/L in DMSO. For in vivo experiments, roniciclib was dissolved in a 40:60 mixture of ethylene glycol/water.

### Cell proliferation (MTS) assay

Cells were seeded into a 96-well plate at a density of 1000–2000 cells per well and incubated overnight. HONE-1 and HK-1 cells were treated in triplicate with serial dilutions of roniciclib and/or cisplatin for 72 h. C666-1 cells were treated for 168 h due to their longer doubling time of ~3.5 days. Control cells were treated with vehicle (0.05% DMSO) only. Cell viability was assessed using an MTS assay (Colorimetric CellTiter 96 Aqueous One Solution Cell Proliferation Assay; Promega, WI, USA). Briefly, the MTS solution was added to each well at a concentration of 1 mg/mL. Following a 2 h incubation, relative cell numbers were quantified by measuring absorbance at 490 nm. IC_50_ values were estimated in GraphPad Prism v4.0 (GraphPad Software Inc., CA, USA). Combinatorial synergistic interactions were tested using the Bliss-independence model.

### Cell cycle and apoptosis (FCM) assay

HONE-1 and HK-1 cells were cultured and treated with vehicle (0.05% DMSO) alone or with varying concentrations of roniciclib for 96 h. C666-1 cells received drug treatment for 240 h owing to their slower replication rate. Cells were harvested, washed with ice-cold PBS, and fixed with ice-cold 70% ethanol for 24 h. The cells were then washed twice with PBS, resuspended at room temperature for 30 min in PI/RNase staining buffer (Annexin V-FITC Apoptosis Detection KIT, BD Pharmingen) containing RNase A at 20 μg/mL, and analyzed on a BD LSRII flow cytometer (BD Biosciences). The fraction of cells in each cell cycle phase was analyzed using FlowJo v10 software (Tree Star Inc., Ashland, OR, USA).

### Western blot analysis

Total protein was harvested from asynchronously proliferating cells in lysis buffer (CellLytic, Sigma-Aldrich) supplemented with ULTA protease inhibitors and PhosSTOP phosphatase inhibitors (Roche Diagnostics, UK). Lysates were sonicated, the supernatant was collected, and protein concentrations were quantified with a BCA Protein Assay kit (Pierce, Rockford, IL, USA). Cell lysates were resolved on 10% SDS-PAGE gels and transferred to 0.2 μm PVDF membranes by western blotting. The membranes were blocked with 5% nonfat dry milk at room temperature for 1 h and then incubated overnight with the following specific primary antibodies: anti-caspase 3 (#9662), anti-PARP (#9542), anti-Survivin (#2803), anti-nucleophosmin (#3542), anti-nucleophosmin phospho-Thr199 (#3541), anti-GAPDH (#2118), and anti-β-actin (#5125) were from Cell Signaling Technology; anti-retinoblastoma phospho-Ser807/801 (#sc-819), anti-MCL-1 (#sc-819), anti-p53 (#sc-126), and anti-Cdc2 (#sc-54) were from Santa Cruz Biotechnology; and anti-RNA polymerase II CTD repeat YSPTSPS Phospho-Ser2 (ab70324) was from Abcam.

### Animal efficacy studies

All animal experiments conformed to the guidelines set forth by the Animal Care and Use Committee of the National University of Singapore, who approved the experimental protocol. Xenografts were generated by subcutaneous injection of 2 × 10^6^ HONE-1 cells into both flanks of BALB/c nude mice. Tumor growth and body weight were measured every 2–3 days. Tumor area was estimated as the product of the longest diameter and its perpendicular diameter. When tumors reached a palpable size of approximately 21 mm^2^, 6 mice were randomized to one of the following treatment arms for 3 cycles: (i) control arm receiving saline i.p. + water/40% PEG p.o.; (ii) cisplatin 6 mg/kg i.p.; (iii) roniciclib 0.5 mg/kg p.o.; (iv) roniciclib 1 mg/kg p.o.; (v) cisplatin 6 mg/kg i.p. + roniciclib 0.5 mg/kg p.o.; or (vi) cisplatin 6 mg/kg i.p. + roniciclib 1 mg/kg p.o. Roniciclib was administered 3 days on/4 days off in monotherapy and 3 days on/11 days off in combination. Cisplatin was administered on day 1 of a 14-day cycle in both the monotherapy and combination regimens. All mice were killed at the end of 42 days.

### Statistical analysis

Data are expressed as mean and standard deviation, and statistical significance of differences was determined by one-way analysis of variance followed by Tukey’s HSD test for multiple comparisons or Student’s paired *t* test for pair-wise comparisons. All statistical tests were two-sided, and *P < *0.05 was considered statistically significant.

## Results

### NPC cells are sensitive to roniciclib treatment at clinically relevant concentrations

We used MTS assay to analyze the effect of roniciclib on the viability of NPC cells. Moderately differentiated (HK-1), poorly differentiated (HONE-1 and CNE-2), and undifferentiated (C666-1) NPC cells were assessed in order to approximate the heterogeneity of tumor cells in vivo. Roniciclib showed antiproliferative activity in HONE-1, HK-1, and CNE-2 cells after 72 h of treatment, with IC_50_ values between 11 and 38 nmol/L. Meanwhile, the Epstein–Barr virus (EBV)-harboring cell line C666-1 exhibited an IC_50_ value of 11 ± 1 nmol/L (Table 1). For downstream analyses, CNE-2 was excluded, as it has been reported to be contaminated with HeLa cells.^15^ To determine whether the sensitivity of cancerous cells to roniciclib might be influenced by the p53 tumor suppressor, which has previously been shown to intercede some of the antiproliferative and tumoricidal effects of CDK inhibitors,^1,2^ we also examined the viability of p53 wild-type (HCT-116-WT) and knockout (HCT-116-p53^−/−^) human colorectal cancer cell lines following roniciclib exposure. However, we did not observe appreciable differences in IC_50_ values (Table 1) between these two cell lines, suggesting that the efficacy of roniciclib is independent of p53 status.

**Table 1:** Cell proliferation assay

**Cell line**	**EBV status**	**P53 status**	**Roniciclib**		**Cisplatin**	
			**Mean IC**_**50**_ **(nM)**	**SD**	**Mean IC**_**50**_ **(μM)**	**SD**
HONE-1	−	Wild type	38.41	4.19	2.75	0.33
HK-1	−	Mutant (exon 5, codon 130, CTC>GTC	28.27	0.50	2.61	0.07
C666-1	+	Mutant (exon 7, codon 249 deletion)	10.97	2.63	9.00	0.94
CNE-2	−	Mutant (exon 8, codon 280, AGA>ACA)	31.02	3.09		
HCT116 +/+	−	Wild type	23.73	3.23		
HCT116 −/−	−	Wild type	25.48	0.98		

Cell lines were treated with roniciclib and cisplatin at various concentrations and the inhibitory concentrations were determined. Mean IC_50_ values were derived from three independent experiments; each concentration was done in triplicates. Respective mean IC_50_'s and standard deviation (SD) were calculated

*IC*_*50*_ half-maximal inhibitory concentration, *SD* standard deviation

### Effects of roniciclib on proliferating NPC cells are consistent with inhibition of multiple CDKs and their substrates

**Fig. 1:**
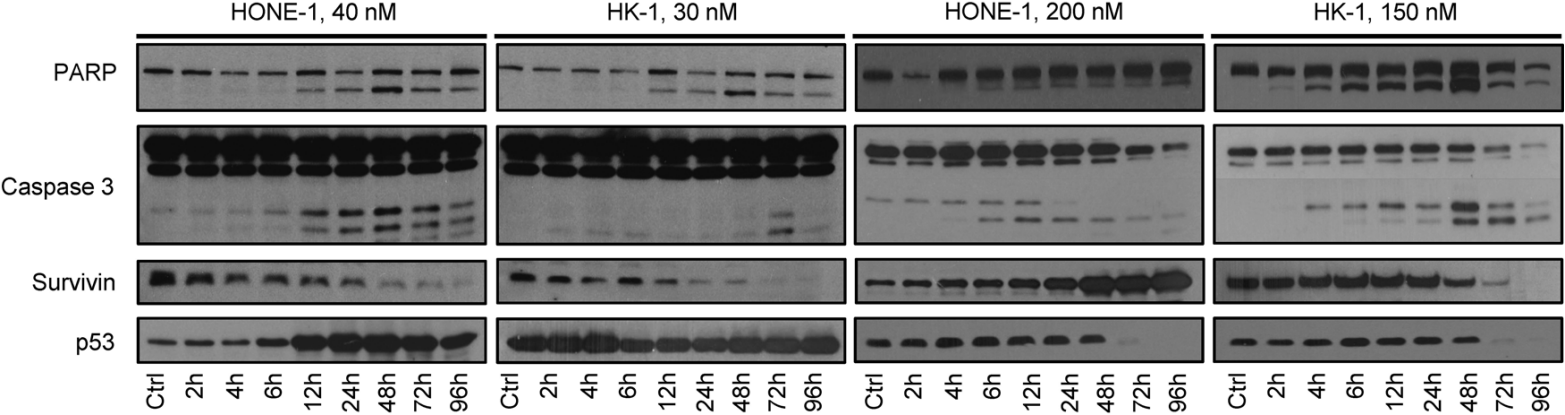
Time- and dose-dependent induction of biochemical changes in HONE-1 and HK-1 cells. Asynchronous NPC cell lines treated with roniciclib at IC_50_ concentrations (HONE-1, 40 nM; HK-1, 30 nM) and five times the IC_50_ concentrations (HONE-1, 200 nM; HK-1, 150 nM) across different time points. Control cells were treated with 0.05% DMSO. Cell lysates were prepared, and 40 µg protein samples were resolved by SDS-PAGE and subjected to western blotting for key apoptotic markers, i.e., PARP, caspase-3, survivin, p53, and GAPDH. All experiments were performed in triplicate

**Fig. 2:**
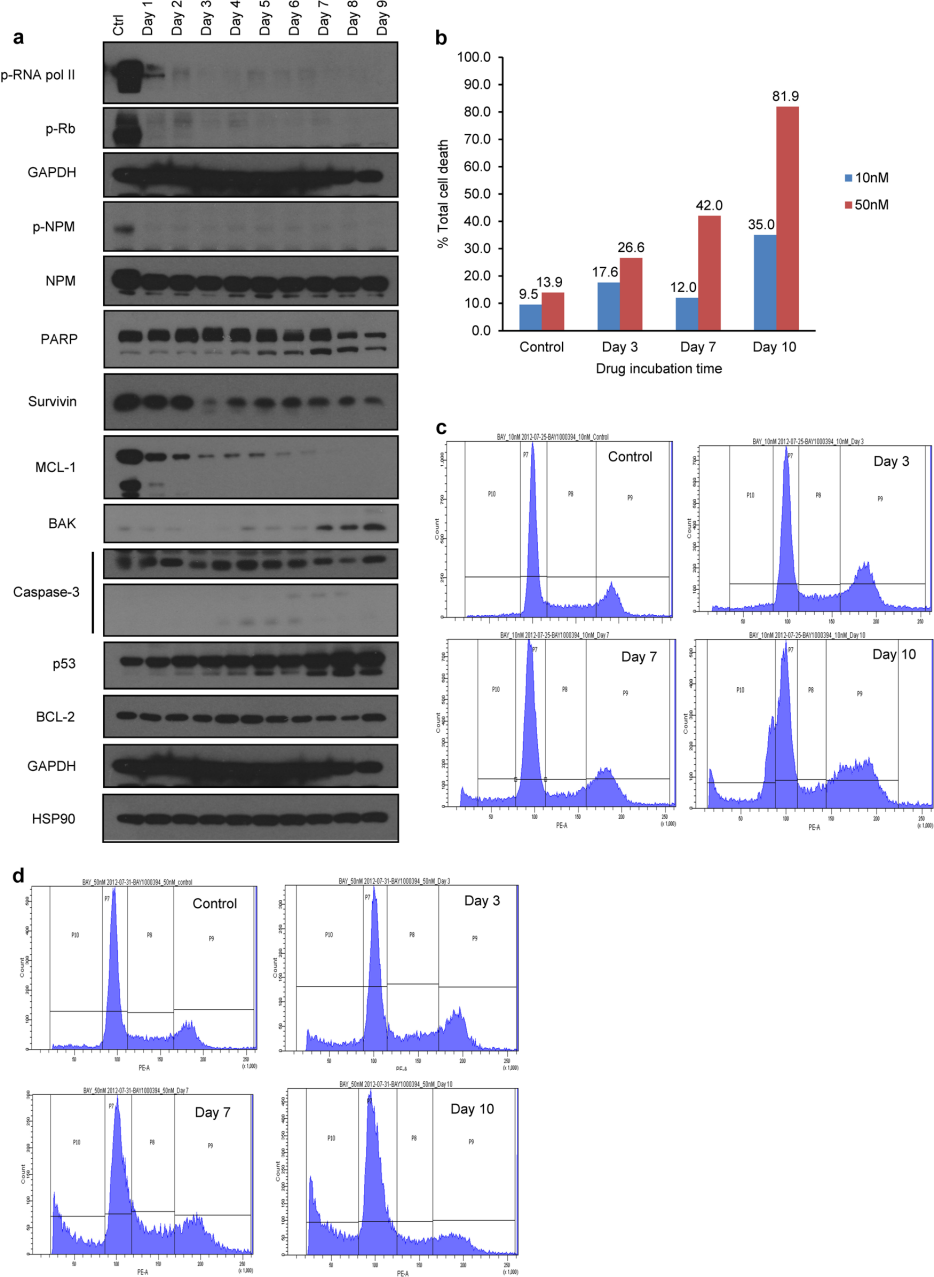
Effects of roniciclib on EBV+ C666-1 cells. **a** Time course and dose-dependent inhibition of direct protein substrates of CDKs in C666-1 cells. Roniciclib significantly inhibited phosphorylation of RNA-polymerase II, retinoblastoma, and nucleophosmin. Drug treatment also induced PARP activation, accompanied by a moderate decrease in survivin expression. The expression of proapoptotic proteins such as BAK, which is known to activate caspases, was enhanced along with concomitant increases in activated caspase-3 and p53 and a decrease in the anti-apoptotic protein MCL-1. **b** Changes in the percentage of dead C666-1 cells over the course of drug incubation. **c** Apoptosis and FCM analysis of C666-1 cells treated at 10 nM. **d** Apoptosis and FCM analysis of C666-1 cells treated at 50 nM. **c** and **d** indicate cell death and cell cycle arrest via G2/M accumulation and subsequent shifts to G0 by days 3, 7, and 10. P7: G1 phase; P8: S phase; P9: G2/M phase; P10: G0 phase. FCM flow cytometry, EBV Epstein–Barr virus

To identify the cellular mechanisms underlying the antiproliferative effects of roniciclib in NPC cells, we assessed apoptosis rates and cell cycle distribution in NPC cell cultures by flow cytometry and western blotting. HK-1 and HONE-1 cells treated with roniciclib at their IC_50_ concentrations (30 and 40 nmol/L, respectively) rapidly underwent apoptosis, as evidenced by increased levels of cleaved PARP (apparent beginning at the 12 h time point) and caspase-3 and a gradual decrease in survivin levels (Fig. 1). Additionally, we observed that p53 expression was significantly increased in HONE-1 cells (but not in HK-1 cells) within 12 h of treatment with 40 or 200 nmol/L roniciclib (Fig. 1). In fact, p53 expression was rapidly attenuated by 48 h in HK-1 cells treated at 150 nmol/L. C666-1 cells treated at their IC_50_ of 10 nmol/L also exhibited a steady rise in p53 expression over 9 days, in accordance with increases in the levels of Bak, activated caspase-3 and cleaved PARP, and a gradual depletion of MCL-1 and survivin (Fig. 2a). However, these results do not contradict our finding that HCT-116 p53 wild-type and knockout mice shared similar IC_50_ readings. Further, these results cumulatively suggest that p53 mutational or activation status does not influence the induction of apoptosis by roniciclib.

**Fig. 3:**
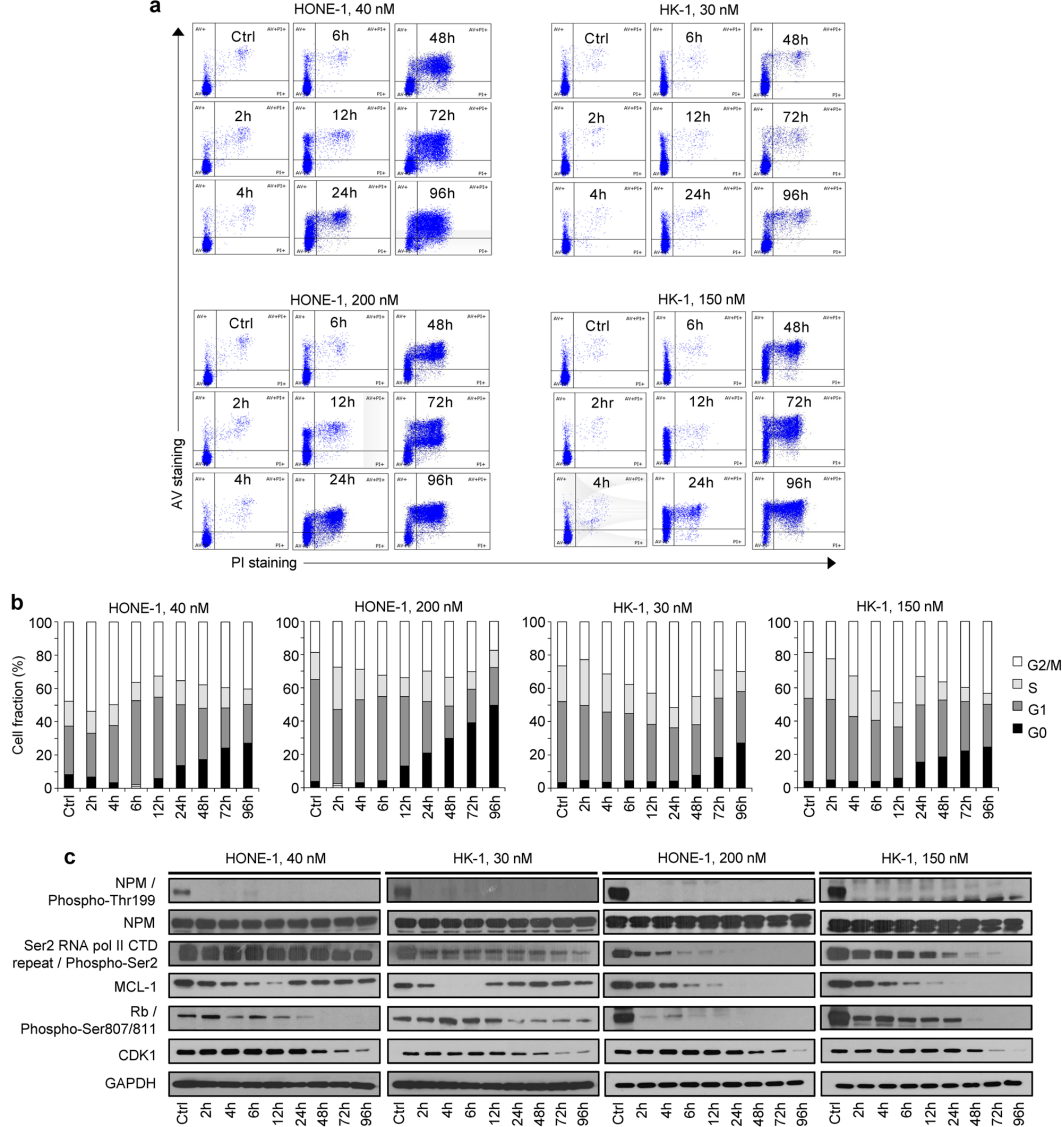
Effects of roniciclib on the cell cycle in HONE-1 and HK-1 cells. **a**–**b** Cell cycle analysis of NPC cell lines treated with roniciclib at two doses at different time points (HONE-1, 40 nM; HK-1, 30 nM; HONE-1, 200 nM; HK-1, 150 nM). Control cells were treated with 0.05% DMSO. Cells were stained immediately with both Annexin V and propidium iodide upon harvesting of cells at the indicated time points and singlet populations were selected via flow cytometry. **c** Time course and dose-dependent inhibition of direct CDK protein substrates in HONE-1 and HK-1 cells. Roniciclib suppressed the expression of p-NPM at both concentrations by 2 h post treatment, suggesting that CDK1 and CDK2/cyclin E expression may be inhibited, as both CDKs are primary targets of NPM. Expression of p-RNA POL II, MCL-1, and p-Rb was downregulated. Higher doses of roniciclib induced rapid Ser-2 dephosphorylation of the C-terminal domain of RNA polymerase II. Dephosphorylation of RNA pol II downregulated transcription, particularly affecting the levels of mRNAs with short half-lives, such as MCL-1. All experiments were performed in triplicate

Flow cytometry indicated that the Annexin V-positive HONE-1 cell fraction was significantly larger 24 h following treatment with 40 nmol/L roniciclib, and over 50% and 80% of cells had undergone apoptosis by 72 h and 96 h, respectively (Fig. 3a) Interestingly, despite exhibiting a comparably pronounced antiproliferative response to roniciclib, the induction of apoptosis in HK-1 cells was less drastic, with less than 30% of the cell population stained with Annexin V/PI by 96 h (Fig. 3a). In C666-1 cells, 35% and 82% of cells treated at 10 nmol/L and 50 nmol/L, respectively, had died by day 10 (Fig. 2b). In terms of cell cycle distribution, there were marginal changes in the proportion of cells in the G1 and S phases; however, a gradual increase in the G2/M subpopulation was observed post-roniciclib treatment in a time-dependent manner (up to 8 h in HONE-1 and 12 h in HK-1 cells). Subsequently, a prominent accumulation of G0 cells was identified simultaneously with a decrease in the G2/M population (Fig. 3b). The accumulation of G0 cells consistently occurred at the same time as an accumulation of Annexin V-stained cells (12 h for HONE-1, 48 h for HK-1), indicating that roniciclib treatment caused cellular apoptosis (Fig. 3a, b). Similarly, cell cycle analysis of C666-1 cells indicated an initial arrest at G2/M and a subsequent accumulation of cells in G0 at days 3, 7, and 10 (Fig. 2c, d).

We next extended the investigation to downstream targets of CDK signaling. Nucleophosmin (NPM), a primary target of CDK1 and CDK2/cyclin E-dependent Thr-199 phosphorylation that is overexpressed in cancer cells and helps to promote cell cycle progression,^16^ was completely inhibited 2 h following roniciclib exposure in HONE-1 and HK-1 cells (Fig. 3c) and by the end of day 1 in the slowly replicating C666-1 cells (Fig. 2a). As cancer cells often display transcriptional addiction to support their rapid growth and survival, it is worth noting that treatment with roniciclib induced rapid Ser-2 dephosphorylation in the C-terminal domain of RNA polymerase II (Fig. 3c), a target of CDK9/cyclin T. Further, roniciclib also suppressed the phosphorylation of retinoblastoma protein (pRb; Figs. 2a and 3c), which implies reduced bioavailability of the transcription factor E2F1.^17,18^ We also examined MCL-1, an anti-apoptotic protein transcribed by RNA polymerase II. At low roniciclib doses, MCL-1 was repressed by 4–6 h, but expression was effectively restored by 12 h in HONE-1 and HK-1 cells (Fig. 3c). In contrast, C666-1 cells treated with low-dose roniciclib exhibited a stable reduction of MCL-1 over a 9-day period (Fig. 2a). Finally, as expected based on roniciclib’s pan-CDK inhibitory activity, the expression of CDK1, which is required for cell cycle progression at G1/S and G2/M,^19^ was markedly diminished 48 h after roniciclib treatment (Fig. 3c). Taken together, these data demonstrate that roniciclib has an antiproliferative effect on NPC cells and acts to prevent cell cycle progression at G1/S phase via Rb, RNA polymerase II, and NPM dephosphorylation and to prevent cell division at G2/M via CDK1 repression, eventually leading to cell death.

### Roniciclib selectively augments the cytotoxicity of cisplatin in NPC cells but not in immortalized cells

Recent data suggest that CDKs play crucial roles in recognizing aberrant DNA structures and activating DNA repair mechanisms, including homologous recombination and non-homologous end joining.^3^ Pharmacological inhibition of CDKs may therefore abrogate DNA damage-induced checkpoint and repair pathways and sensitize cancer cells to DNA-damaging chemotherapy. We therefore investigated the utility of combinatorial approaches involving roniciclib and cisplatin, a genotoxic agent which is currently used in front-line chemoradiotherapy regimens for locoregionally advanced and metastatic disease.^13^

**Fig. 4:**
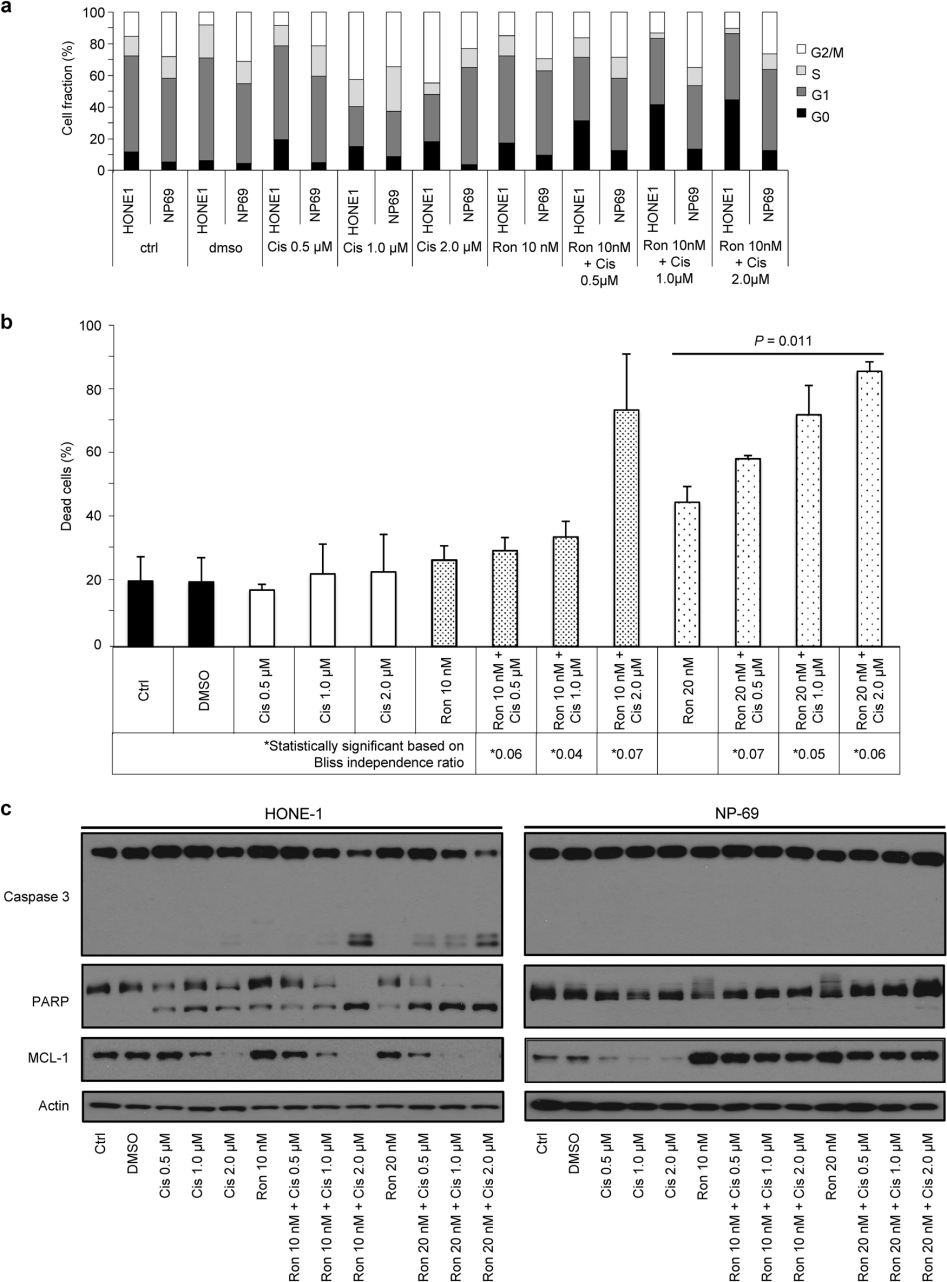
Effects of roniciclib–cisplatin combinations in vitro. **a** Cell cycle analysis of combined roniciclib- and cisplatin-treated HONE-1 and NP69 cells. NP69 is an immortalized nasopharyngeal epithelial cell line. **b** HONE-1 and NP69 cell lines were treated at the indicated doses for 72 h. Protein expression levels of caspase-3 and PARP were used as indicators of apoptosis. Synergistic effects of both drugs were observed in the undifferentiated NPC cell line (HONE-1) but not in the nasopharyngeal epithelial cell line. (*) denotes a synergistic effect of both drugs based on the Bliss independence ratio. **c** Dual stains (Annexin and propidium iodide) were applied to HONE-1 cells treated with both roniciclib and cisplatin. All experiments were performed in triplicate

HONE-1 and HK-1 cells were treated with combinations of roniciclib and cisplatin at effective concentrations below their respective 50% inhibition responses for 72 h, and then combinatorial synergistic interactions were tested using the Bliss-additivity model (Supplementary Table S1). Treatment with 10 nmol/L roniciclib combined with 2.0 μmol/L cisplatin resulted in greatly enhanced cell death; this effect was over 250% greater than that achieved by either drug alone (Fig. 4a, b). Likewise, the combination of 20 nmol/L roniciclib with 0.5, 1.0, or 2.0 μmol/L cisplatin produced a highly synergistic apoptotic effect (Fig. 4a, b), supported by an increase in cleaved caspase-3 and PARP (Fig. 4c). Complete inhibition of MCL-1 was also achieved with low-dose combinations of roniciclib and cisplatin (Fig. 4c), while 200 nmol/L roniciclib alone was required for complete MCL-1 inhibition (Fig. 3c). However, these same effects were not seen in immortalized NP69 cells (Fig. 4c).

### Roniciclib is active as a monotherapy and synergizes with cisplatin in NPC xenografts

**Fig. 5:**
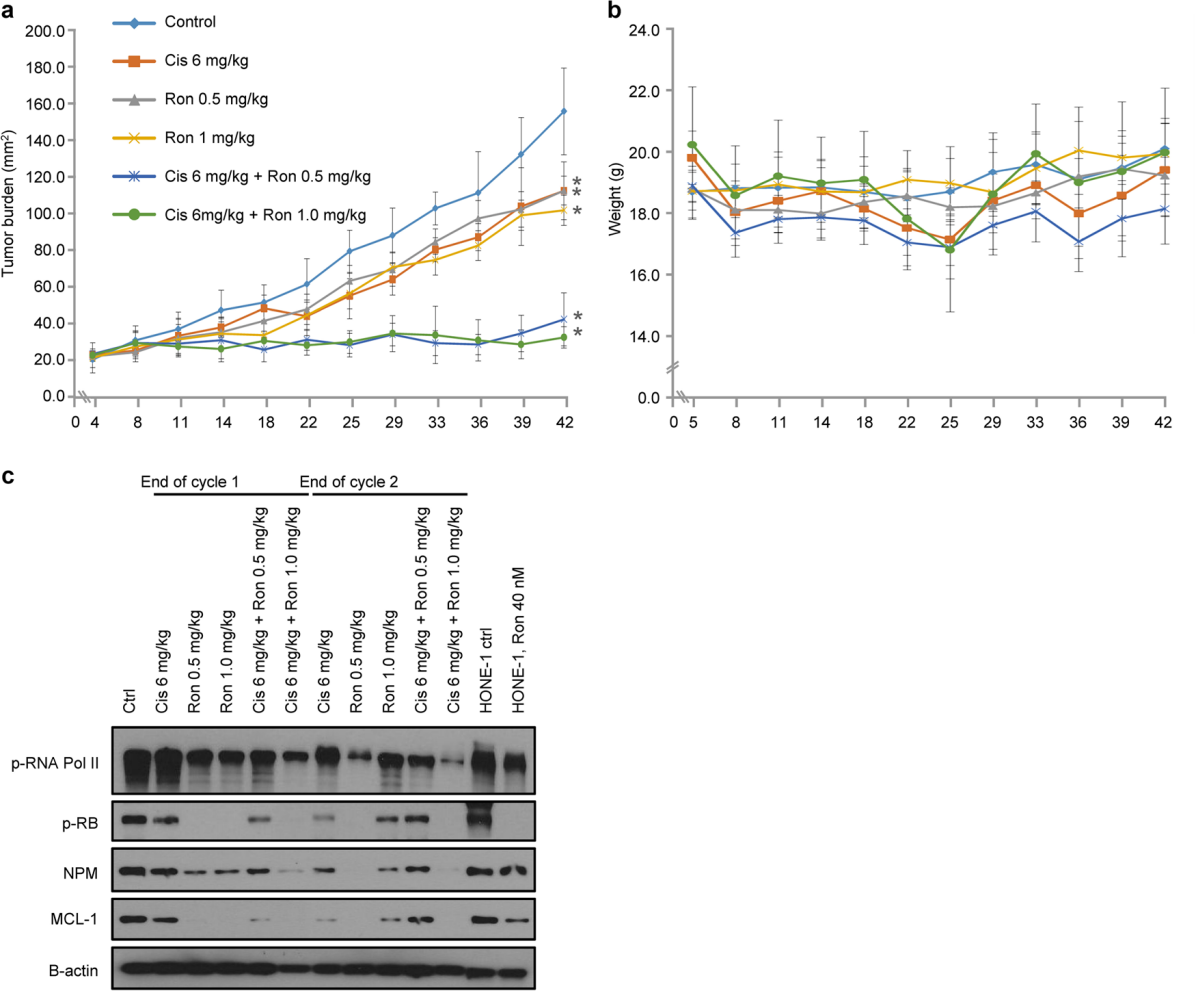
Effects of roniciclib–cisplatin combinations in vivo. **a** BALB/c nude mice were subcutaneously injected with HONE-1 cells. Dosing schedules are shown in the figure. **b** Mouse weights (g) were monitored throughout the study. (*) denotes statistical significance (*p* value <0.05). **c** Protein expression of direct cell cycle substrates on mouse tumor tissues harvested 24 h after the first and second treatment cycles, respectively. BALB/c nude mice were subcutaneously injected with HONE-1 cells. Mice were treated at 0.5 mg/kg and 1 mg/kg twice daily every 3 days on/4 days off

To investigate the in vivo therapeutic effects of roniciclib and the roniciclib–cisplatin combination, we utilized BALB/c xenograft mice subcutaneously implanted with HONE-1 cells. Tumors were allowed to grow to ~21 mm^2^ before the mice were randomized into groups receiving roniciclib as a monotherapy, roniciclib in combination with cisplatin, or control. Tumor measurements at day 42 showed that 0.5 mg/kg and 1 mg/kg roniciclib resulted in significant suppression of tumor growth (56.85–63.8% and 57.03–72.27%, respectively) (Fig. 5a). Although single-agent roniciclib or cisplatin demonstrated remarkable tumor growth inhibition compared to control, the combination regimens displayed greatly enhanced efficacy in suppressing tumor growth (Fig. 5a). One in eight mice treated with 1 mg/kg roniciclib and 6 mg/kg cisplatin lost >20% of its bodyweight and was killed as per ethical guidelines, while all other mice tolerated the prescribed regimens without gross toxicity (Fig. 5b).

We also investigated the in vivo effects using protein assays. A randomly selected mouse from each treatment arm was humanely killed, and tumors were harvested at the end of the first and second cycles. Figure 5c shows changes in the expression levels of RNA polymerase II, Rb, NPM, MCL-1, and β-actin compared to control. Not surprisingly, the levels of phosphorylated protein were lower in mice receiving treatment compared to controls. However, these effects were more subdued in mice that received 6 mg/kg cisplatin + 1 mg/kg roniciclib. Taken together, these data demonstrate that roniciclib has potent anti-NPC activity in vivo as either a monotherapy or a combination treatment.

## Discussion

Although locoregionally advanced nasopharyngeal carcinoma (NPC) is considered relatively curable with concurrent chemoradiotherapy,^20^ survival rates have plateaued in part because novel targeted therapies have yet to encroach on the current therapeutic armamentarium. In the recurrent and metastatic settings, the median survival is 12–20 months with aggressive multimodal therapy,^13^ emphasizing the need for novel treatment strategies. As with most cancers, the cyclin-dependent kinases (CDKs) and their cyclin partners are eminently deregulated and hyperactive in NPC and have been shown to mediate an aggressive clinical phenotype and confer worse prognoses.^21,22^ For instance, we and others have previously reported that genetic alterations leading to hyperactivation of cyclin D1 signaling, such as allelic deletion of its negative regulator p16^INK4A^ and *CCND1* amplification and/or overexpression, are highly recurrent somatic events and drivers of tumorigenesis in primary NPC.^23–29^ Furthermore, our earlier work with seliciclib, a selective inhibitor of CDKs 2, 7, and 9, demonstrated that CDK inhibition may represent a valuable therapeutic strategy in NPC, mediating tumor responses in 7 of 14 patients.^14^

In the present study, we found that the novel pan-CDK inhibitor roniciclib (BAY1000394) displays potent anti-NPC efficacy, which is further enhanced by co-administration with cisplatin in vitro and in vivo. Given that roniciclib avidly inhibits the activities of cell cycle CDKs 1, 2, 3, and 4 and transcriptional CDKs 7 and 9 at the nanomolar concentrations used in our experiments, it is not surprising that tumor cell death may be effected through pleiotropic mechanisms. Pharmacological blockade of CDKs 1 and 2 was attained, as demonstrated by the suppression of one of their primary targets, nucleophosmin, which itself plays an important role in promoting G1/S and G2/M phase progression.^16^ Treatment with roniciclib also repressed the transcriptional apparatus, as substantiated by rapid Ser-2 dephosphorylation in the C-terminal domain of RNA polymerase II, which is a target of CDK 9/cyclin T. The downregulation of cancer cell transcriptional activities is likely responsible for the diminished expression of MCL-1, an anti-apoptotic protein, as well as CDK1, which is also required for the G1/S and G2/M transitions. Furthermore, NPC cell lines displayed reduced phosphorylation of the retinoblastoma tumor suppressor protein, which would result in lower levels of the transcription factor E2F1 and consequently lead to G1 arrest and/or cellular senescence. Therefore, in summary, our data suggest that roniciclib’s mechanisms of action may be attributed to (1) targeting of the transcriptional apparatus which sustains the biological processes driving rapid growth and proliferation, (2) interfering with the balance of pro- and anti-apoptotic factors, and (3) directly eliciting cell cycle blockade.

Crucially, we also observed that roniciclib treatment selectively chemosensitized NPC cells but not untransformed NP69 cells to cisplatin. One explanation for this phenomenon is that the overexpression of both CDK1 and CDK2 in the malignant state increases the ability of CDK2 to compensate for CDK1 loss. CDK2 compensation may enable transformed cells to proliferate despite CDK1 inhibition, thus enhancing their sensitivity to S-phase DNA-damaging agents.^30,31^ In contrast, inhibition of CDK1 in untransformed cells rapidly ushers in G2/M arrest and DNA repair and antagonizes the response to subsequent DNA damage.^3,11^ Importantly, this mode of action may also offer a valuable strategy for counteracting the development of cell cycle-mediated chemoresistance.^32^ Alternatively, it is also possible that selective killing of transformed cells is achieved via cyclin A/CDK 2 inhibition, whereby higher baseline levels of E2F1 activity in transformed cells persist during S-phase transversal^33^ and serve as an apoptotic signal.^34^

Recently, two parallel first-in-human phase I studies of roniciclib in various advanced malignancies have been reported.^7–9^ It is worth noting that neither phase I study of roniciclib enrolled any patients with NPC.^7–9^ Nevertheless, these studies have established that single-agent roniciclib is well tolerated as a 3 days on/4 days off regimen, which is consistent with the absence of overt weight loss seen in our xenograft murine model treated with 0.5 mg/kg on the same dosing schedule. In these early phase trials, the most common adverse events included nausea, fatigue, diarrhea, and vomiting, consistent with the on-target effects of CDK inhibition, which invariably affects the rapidly cycling enterocytes of the intestinal lining. Clinical trials of other CDK inhibitors, such as the third-generation selective CDK4/6 inhibitor abemaciclib, have also incurred similar rates of gastrointestinal toxicities, with the incidence of all-grade diarrhea approaching ~90% in recent clinical trials of the third-generation selective CDK4/6 inhibitor abemaciclib.^35,36^ Interestingly, unlike other CDK inhibitors, roniciclib does not appear to have a marked effect on neutrophils.^7–9^ This could be related to the intermittent dosing schedule or to exposure levels below the hemotoxic concentration.

Platinum-containing chemotherapy represents the cornerstone of first-line systemic treatments for NPC.^13^ Hence, our finding that roniciclib selectively potentiates cisplatin cytotoxicity in NPC cells without affecting untransformed cells is of clinical relevance. Taken together with recently reported safety results,^7–9^ these data would seem to suggest that concurrent administration of roniciclib and cisplatin may lead to better disease control without incurring significant additional toxicities, thus improving the overall therapeutic index for concurrent or adjuvant chemotherapies. Concentrations expected to deliver meaningful pharmacological activity should be clinically attainable. In our experiments, roniciclib restrained NPC cell proliferation and induced apoptosis at IC_50_ values ranging from 11 to 38 nmol/L and effectively curbed tumor growth in murine xenograft models when administered as a monotherapy at a dose of 0.5 mg/kg.

In conclusion, these findings provide strong support for evaluating the efficacy of roniciclib in patients with NPC and lay the groundwork for combination studies with cisplatin. In the future, it will be interesting to ascertain the combination effects and to establish the optimal sequence and schedule of roniciclib with concurrent radiotherapy and chemotherapeutic agents, including gemcitabine, capecitabine, vinorelbine, and irinotecan, which are used as second-line treatments for recurrent and metastatic disease. Finally, it may be worth exploring combinations of roniciclib with novel immunotherapies, as CDK inhibitors have recently been shown to also promote tumor immunogenicity and synergize with immune checkpoint blockade.^37,38^

## Electronic supplementary material

Supplementary Table 1
