# Supplementary material for: Pan-CDK inhibition augments cisplatin lethality in nasopharyngeal carcinoma cell lines and xenograft models
Source: Signal Transduct Target Ther. 2018 Apr 13;3:9. doi: 10.1038/s41392-018-0010-0 (PMC5897350; doi:10.1038/s41392-018-0010-0)
Supplement: Supplementary file 1 — Supplementary Table 1 [file 41392_2018_10_MOESM1_ESM.docx]

| **Supplementary Table S1. Bliss Independence Calculations** | | | | | | | | | | |
| --- | --- | --- | --- | --- | --- | --- | --- | --- | --- | --- |
| **Cell Line** | **BAY1000394** | | | **Cisplatin** | | | **Combination therapy** | | **Bliss independence (Fractional survival)** | **Interpretation** |
| HONE-1 | Drug dose (nM) | Survival | S.D | Drug dose (nM) | Survival | S.D | Survival | S.D |  |  |
|  | 9.60 | 0.994 | 0.032 | 687.25 | 0.910 | 0.044 | 0.871 | 0.015 | 0.962 | additivity |
|  | 19.21 | 0.825 | 0.037 | 1374.50 | 0.743 | 0.025 | 0.476 | 0.023 | 0.777 | synergism* |
|  | 38.41 | 0.202 | 0.034 | 2749.00 | 0.394 | 0.015 | 0.076 | 0.004 | 0.951 | additivity |
|  | 76.82 | 0.140 | 0.006 | 5498.00 | 0.153 | 0.018 | 0.098 | 0.002 | 4.588 | antagonism |
|  | 153.64 | 0.165 | 0.004 | 10996.00 | 0.025 | 0.002 | 0.093 | 0.002 | 22.943 | antagonism |
|  | 38.41 | 0.199 | 0.019 | 2.749 | 1.014 | 0.077 | 0.136 | 0.007 | 0.673 | synergism* |
|  | 38.41 | 0.199 | 0.019 | 0.002749 | 1.039 | 0.032 | 0.152 | 0.008 | 0.733 | synergism* |
| HK-1 | 7.068 | 0.959 | 0.011 | 653.000 | 0.966 | 0.022 | 0.879 | 0.018 | 0.949 | additivity |
|  | 14.135 | 0.785 | 0.040 | 1306.000 | 0.882 | 0.007 | 0.629 | 0.015 | 0.908 | additivity |
|  | 28.270 | 0.311 | 0.003 | 2612.000 | 0.593 | 0.008 | 0.204 | 0.011 | 1.107 | antagonism |
|  | 56.540 | 0.061 | 0.005 | 5224.000 | 0.272 | 0.025 | 0.009 | 0.001 | 0.554 | synergism* |
|  | 113.080 | 0.016 | 0.001 | 10448.000 | 0.054 | 0.007 | 0.002 | 0.001 | 2.057 | antagonism |
|  | 28.270 | 0.337 | 0.013 | 2.612 | 0.969 | 0.019 | 0.099 | 0.004 | 0.303 | synergism* |
|  | 28.270 | 0.337 | 0.013 | 0.003 | 0.962 | 0.018 | 0.088 | 0.004 | 0.270 | synergism* |
|  |  |  |  |  |  |  |  |  |  |  |
